# Climate change, health and environmental policy in Africa: structural constraints, regional disparities and global inequities

**DOI:** 10.3389/fpubh.2026.1816888

**Published:** 2026-06-30

**Authors:** Silvany Simone Tavares, Cindy J. Xie, Amulya Aluru, Illyane Sofia Lima, Ana Catarina Pêgo, Raffaella Gozzelino

**Affiliations:** 1 Inflammation and Neurodegeneration Laboratory, NOVA Medical School Research, NOVA University of Lisbon, Lisbon, Portugal; 2 Department of Urban Studies and Planning, Massachusetts Institute of Technology, Cambridge, MA, United States; 3 Diáspora Mundi, Praia, Cape Verde; 4 Department of Electrical Engineering and Computer Science, Massachusetts Institute of Technology, Cambridge, MA, United States

**Keywords:** Africa, climate change and health, climate justice and equity, food security and nutrition, vector-borne diseases

## Abstract

Africa experiences a disproportionate burden of climate-related health impacts. Yet it contributes minimally to cumulative global greenhouse gas emissions. Rising temperatures, ocean acidification, sea level rise, ecosystem degradation, and environmental pollution increasingly shape food insecurity, infectious disease risk, and population wellbeing. In the last decade, global policies have advanced ambitious agendas to mitigate the impacts of climate change. However, those strategies are mainly defined by high-income countries, which depend on institutional, infrastructural, and financial capacities that are unevenly distributed between the so-called “Global North” and “Global South.” Hence, the direct transfer of global policies to African settings often misaligns with local realities, reinforcing vulnerabilities rather than promoting resilience. This review provides an African perspective of climate-related health impacts, highlighting structural constraints that compromise the capacity of this continent to effectively respond. The increasing prevalence of food insecurity, nutritional deficits and vector-borne disease outbreaks reveals fundamental differences in the buffering capacity and the feasibility of climate strategy implementation in relation to advanced economies. The need to formulate and operationalize policy frameworks grounded in African realities becomes essential to protect population health, enable effective adaptation to climate-related alterations and impact, in addition to support the sustainable development of this continent.

## Introduction

Climate, environmental and health policies are largely shaped by high-income countries, which often limit their relevance in settings where infrastructure, institutional capacity, and financial resources remain constrained, particularly in low and middle-income regions (1, 2). The global transfer of these standards, therefore, risks exacerbating inequities rather than strengthening resilience (3). Current international policy frameworks, many of which are based upon assumptions that cannot be presumed across African settings, exacerbate these misalignments. Policies that depend on the implementation of stable regulatory enforcements, reliable surveillance platforms, broad fiscal space, digitalized administrative systems, and universal health service coverage, are unrealistic when foundational systems, such as water, sanitation, transport, energy reliability, scientific services and/or primary healthcare coverage are incomplete. In those cases, direct policy transfer results in shifting scarce public resources at country's disposal toward the compliance of indicated targets, but failing to reduce population vulnerability.

Major international assessments have established climate change as a central determinant of global health (4–6). However, the ability to cope with climate-related health impacts is unevenly distributed, dividing the world into a so-called “Global North,” with substantial adaptive capacity, and a “Global South,” where the syndemic challenges of environmental exposure, susceptibility, and resource limitations converge (7–9). Global climate stressors produce markedly unequal health outcomes across countries and regions, underscoring the need for equity-oriented strategies that bridge the gap between policy ambition and implementation feasibility (10). These disparities are not only economic, but also epistemic and institutional. Data used to shape climate policy are disproportionately collected and analyzed in high-income settings, where hazard profiles, labor structures, housing conditions, insurance coverage, and health systems buffers differ significantly from those found in Africa. Effective interventions in advanced economies underperform when transferred indiscriminately to contexts characterized by informal employment, rapid urbanization, climate-sensitive livelihoods, and higher baseline burdens of infectious disease and undernutrition.

Africa has contributed minimally to environmental degradation, which has been widely recognized as a major determinant of human health (11, 12). This difference in contribution is evident in metrics of cumulative greenhouse gas emissions, which advanced economies compensate for by carbon offsetting and externalizing production (13, 14) (Table 1). Yet under current international frameworks, Africa is expected to safeguard ecological stability and practice sustainable economic development, while facing severe constraints in the resources required to mitigate the escalating health risks associated with extreme climate events (4, 5). These pressures are further aggravated by conditions of extreme poverty, rapid demographic growth, and fragile health systems (6, 8). Given this historical context, integrating a consideration of climate justice into global environmental policies is crucial to enabling a just transition across the region. Climate justice provides a necessary ethical framework. It links different responsibility for emissions with distinct capacity to respond, while calling for a fair distribution of burdens, resources, and decision-making influence. In Africa, climate justice extends beyond financial transfers. It also includes equitable access to technology, adaptation finance, scientific collaboration, and policy voice, without which global environmental governance risks to function as an unfunded mandate for countries least responsible for the crisis (15).

**Table 1:** A comparison of targeted vs. actual greenhouse gas (GHG) emissions in African countries, expressed in kilotons of CO_2−_equivalent (kt CO_2_eq).

Region	Country	Latest NDC	Targeted emission goals (as stated in nationally determined contribution document)	2024 Emissions (kt CO_2_eq)	Percentage of 2024 global emissions
Northern Africa	Algeria	2015	“A greenhouse gas emission reduction of 7% to 22% by 2030 compared to business as usual (BAU) levels, conditional on external assistance for the financing of the development and transfer of technologies and capacity building. Seven percentage GHG reduction will be achieved with domestic means”	252,700.0	0.475%
	Egypt	2020	“Egypt commits to reducing its electricity emissions by 37%, its oil and gas emissions by 65%, and its transport emissions by 7% by 2030 relative to BAU”	386,598.2	0.727%
	Libya	*N/A*	*No document submitted*	98,170.9	0.185%
	Morocco	2025	“An unconditional net reduction in GHG emissions across the economy of 21.6% in 2035 compared to the baseline scenario (Normal Business Course), with the country's own resources, and secure international support to date. A conditional net reduction in economy-wide GHG emissions of 31.4% in 2035 compared to the Reference Case (Normal Business Course), thus raising the national ambition to reduce GHG emissions to 53% in 2035 compared to the Reference Case (NAC)”	108,736.4	0.204%
	Tunisia	2025	“Tunisia's unconditional contribution corresponds to a reduction of carbon intensity by 31% in 2035 compared to the reference year 2010. The conditional contribution provides for an additional reduction of carbon intensity by 31% in 2035 compared to 2010, bringing the total reduction in carbon intensity to 62% by 2035 compared to 2010”	44,621.0	0.084%
Eastern Africa	Burundi	2025	“The goal of NDC 3.0 is to reduce greenhouse gas emissions by 23% by 2035 compared to the baseline scenario (BAU), which corresponds to a 3% reduction under its unconditional target and a 20% reduction under its conditional target. Targets with and without measures have been defined by sector (Energy, PIUP, Agriculture, FAT, and Waste)”	8,421.7	0.016%
	Comoros	2020	“The Union of the Comoros forecasts, through this revised NDC, a net reduction in these GHG emissions, excluding LULUCS, of 23% and an increase in its net sink ofCO2 removals of 47% by 2030 compared to the reference scenario. The overall cost of this ambition is estimated at 902 million euros, of which 96 million euros is unconditional or 5%”	949.4	0.002%
	Djibouti	2015	“The target in relation to the benchmark indicator is 65% reduction divided between two (02) scenarios, namely the unconditional scenario at 41.30% and the conditional scenario at 23.70%”	2,417.3	0.005%
	Eritrea	2025	“The combined mitigation target (unconditional and conditional elements) corresponds to a reduction of 24.4 percent compared to the BAU projection by 2030. The unconditional component foresees emission reductions of 8.6 percent and the conditional component contributes to approximately 15.8 percent compared to the BAU projection by 2030”	6,579.3	0.012%
	Ethiopia	2025	“The NDC 3.0 commits to reducing greenhouse gas (GHG) emissions by 70.3% by 2035, compared to a revised business-as-usual pathway, a slight increase from the earlier 68.8% target”	192,704.0	0.362%
	Kenya	2025	“Kenya aims to implement mitigation measures to achieve absolute emission reductions of 75 MtCO2eq by 2035. Out of the 75 MtCO2eq, unconditional measures are expected to lead to a 15 MtCO2eq emission reduction amount. An additional 60 MtCO2eq emission reduction is achievable if support is made available from international sources to cover the full cost for implementation”	102,836.2	0.193%
	Madagascar	2025	“By 2030, NDC2 aims to reduce greenhouse gas emissions by 28%, or 48,403 Gg eq. CO2. In addition to this reduction in emissions, NDC2 aims to increase its	35,790.0	0.067%
			greenhouse gas absorption capacity by around 20%, i.e., −37,809 Gg eq. CO2 from additional sequestration”		
	Mauritius	2025	“…aims to reduce overall GHG emissions by 40% in 2035 compared to the Business as Usual (BAU) scenario of about 7385 Gg CO2e including Land Use, Land Use Change and Forestry (LUCF” title = “Land-Use, Land-Use Change and Forestry” >LULUCF) in 2035. This economy-wide emissions reduction target comprises sector specific mitigation targets for energy, transport, waste, Agriculture, Forestry and Other Land Use (AFOLU) and IPPU”	6,471.6	0.012%
	Rwanda	2025	“Unconditional contribution: A reduction of 7 per cent (%) relative to BAU in the year 2035; equivalent to an estimated mitigation level of 1.84 million tons of carbon dioxide equivalent (tCO2e) in that year. This is an unconditional target, based on domestically supported and implemented mitigation policies and measures. Conditional contribution: An additional reduction of 46% relative to BAU in the year 2035, equivalent to an estimated additional mitigation level of 13.02 million tCO2e in that year. This represents an additional contribution, conditional on the provision of international support and funding”	8,349.9	0.016%
	Seychelles	2025	“Emission reduction target is 23.1% (204 ktCO2e reduction) relative to business-as usual emissions in 2035. This constitutes an increase in ambition compared to the Seychelles' NDC 2.0, as it will allow achieving a reduction of 35.7% compared to the BAU for 2030, whereas in the NDC 2.0 the WEM was projected to be 26.4% below BAU”	976.4	0.002%
	Somalia	2025	“As a result, the country's mitigation contribution takes the form of both conditional and unconditional reduction in GHG emissions relative to a business-as-usual (BAU) emissions baseline over the period 2025 to 2035. Total emission reduction target is 34%, with 29% under conditional reduction while 5% under unconditional”	34,416.9	0.065%
	South Sudan	2020	“After finalizing the sectors to be included in the NDC, a detailed assessment of each sector was conducted. This assessment, along with the outputs of the material flow analysis, was used to develop mitigation and adaptation strategies for each individual sector...In total, by implementing these strategies South Sudan can reduce an estimated 109.87 million tons of carbon dioxide equivalent (tCO2e) and sequester 45.06 million tCO2e by 2030”	*Data not available*	*Data not available*
	Sudan	2020	“The updated Sudan NDC defines mitigation targets as fixed level GHG emission reductions in 2030 relative to the BAU level in that year, as summarized below: Energy: 12,458,008 tCO2e Forestry: 13,384,246 tCO2e Waste: 1,278,822 tCO2e”	138,702.0	0.261%
	Uganda	2020	“Economy wide emissions reduction of 24.7% in 2030 below the BAU conditions. Of which, Uganda's unconditional efforts will result into reduction of 5.9% in 2030 below the BAU conditions”	57,298.2	0.108%
Southern Africa	Angola	2025	“Angola sets itself the goal of achieving this 5 % reduction in emissions by 2035, unconditionally. In addition, it is hoped that, through a conditional mitigation scenario, the country will be able to reduce 11% below BAU emission levels by 2035”	69,922.9	0.131%
	Botswana	2025	“The projected GHG emissions under the mitigation scenario represent avoided GHG emissions of approximately 15% by the year 2030. The updated NDCs have identified 26 quantifiable mitigation actions from three key sectors: energy (stationary and mobile), IPPU, and AFOLU”	12,097.7	0.023%
	Lesotho	2025	“…Lesotho is committed to an unconditional reduction of GHG emissions of 6 % (419 ktC02eq) from the BAU by the year 2030. Further, the government of the Kingdom of Lesotho commits to an additional conditional reduction of GHG emissions of 18 % (1,270 ktC02eq) from the 2021 baseline by the year 2030, provided that commensurate international support is received in the form of finance, investment, technology development and transfer, and capacity building to cover the full cost of implementing proposed additional mitigation actions”	3,055.0	0.006%
	Malawi	2020	“Unconditional contribution: a reduction of 6 per cent relative to BAU in the year 2040; equivalent to an estimated mitigation level of [2,100 ktCO2e] in that year. This is an unconditional target, based on domestically supported and implemented mitigation measures and policies. Conditional contribution: An additional reduction of 45% relative to BAU in the year 2040; equivalent to an estimated mitigation level of [15,600 ktCO2e]…This represents an additional targeted contribution, based on the provision of international support and funding”	19,023.6	0.036%
	Mozambique	2025	“The target year is 2035. The emissions reduction target is under development and will be followed by extensive technical review and consultations. An emissions reduction target of between 15 and 25% of baseline is envisaged (BUR2 2020)”	33,625.6	0.063%
	Namibia	2020	“Namibia, despite being historically a sink and projected to remain so by the target year 2030 in the BAU scenario started to undertake mitigation as from the base year 2010 and is committed to continue on this path to reduce its emissions. A total mitigation potential of [11,902 ktCO2e] in absolute terms is projected, representing an increase in the sink capacity by 13.1% compared to the BAU scenario in 2030”	14,212.1	0.027%
	South Africa	2025	“South Africa's annual…emissions will be in a range from [320,000 - 380,000 ktCO2e] in 2035”	569,809.6	1.071%
	Eswatini	2025	“Eswatini's new economy wide target, compared to the Business as Usual (BAU) scenario, aims to reduce its greenhouse emissions (GHG) by 2.24 MtCO_2_eq in 2035. This is an increase of 115% from the 1.04 MtCO_2_eq reductions by 2030 in NDC 2.0. Subject to national circumstances, Eswatini aims to mobilize domestic resources to abate 695 ktCO_2_eq tons of GHG emissions (31% of the 2.24 million tons). The reduction of the remaining 1.55 MtCO_2_eq tons (69% of the total) will be achieved through a combination of international support, including finance, investments, technology development and transfer, and capacity building”	2,929.8	0.006%
	Tanzania	2020	“Tanzania will reduce greenhouse gas emissions economy-wide between 30%−35% relative to the Business-As-Usual (BAU) scenario by 2030, whereby about [138,000–153,000 ktCO2e] gross emissions is expected to be reduced, depending on the baseline efficiency improvements, consistent with its sustainable development agenda. The emissions reduction is subject to review after the First Biennial Update Report (BUR) and Updated GHG inventory”	102,525.0	0.193%
	Zambia	2025	“Zambia intends to reduce its greenhouse gas emissions by 25% [at Business As Usual (BAU) level of international support prevailing in 2015] and toward 47% (with substantial international support) by 2030”	38,519.0	0.072%
	Zimbabwe	2025	“…the country revised NDC2.0 to raise its ambition to reduce GHG emissions by at least 40% per capita in the NDC3.0 compared to the 2035 Business-as Usual (BAU) scenario”	30,784.3	0.058%
Western and Central Africa	Benin	2020	“For the coming years, the measures envisaged in the revised NDC, in the Energy, Agriculture and Waste sectors are likely to contribute to reducing cumulative GHG emissions (excluding Land-Use, Land-Use Change and Forestry) by around 48.75	17,767.1	0.033%
			MtCO2e compared to the reference scenario, i.e., a reduction of around 20.15% over the period 2021–2030”		
	Burkina Faso	2025	“NDC 3.0 significantly raises GHG emission reduction targets for 2030 and 2050 and aligns with the Long-Term Low-Carbon and Climate-Resilient Development Strategy (LT-LEDS). In the mitigation component, the country is committed to reducing its greenhouse gas emissions by 30.87% by 2030 compared to the baseline scenario, i.e., 26,241.81 Gg CO2 eq. This effort is made up of 22.37% unconditional shares and 8.50% conditional shares. The contributing sectors are (i) Agriculture, (ii) Forestry and Other Land Use (FAT), (iii) Energy, (iv) Transport, (v) Industrial Processes and Product Use (IPU), (vi) Waste and WASH. For the adaptation component, it is planned to reduce GHG emissions by 41.94% (i.e., 35,651.32 Gg CO2 eq) by 2030 with 39.67% of unconditional actions. The sectors targeted by adaptation measures are Agriculture, TF, water resources, livestock, waste, and WASH, housing and infrastructure. Beyond 2030, the overall ambition to reduce GHG emissions reaches 55.23% in 2035 and 75.55% in 2050 for mitigation, and 70.31% in 2035 and 84.40% in 2050 for adaptation”	34,917.7	0.066%
	Cameroon	2020	“Reduction of GHG emissions by 35% compared to a reference scenario for the target year 2030 and divided into 12% unconditional and 23% conditional on the support of the international community in the form of financing, capacity building actions and technology transfer”	40,621.9	0.076%
	Cabo Verde	2025	“By 2030, Cabo Verde commits to reduce economy-wide GHG emissions by 18% below the BAU scenario (unconditional). Conditional on adequate international support, this reduction target may increase to 28% below BAU. By 2035, mitigation ambition rises to a 39% reduction below BAU (conditional), while maintaining 18% below BAU (unconditional)”	912.6	0.002%
	Central African Republic	2020	“The mitigation measures taken will generate, according to the unconditional scenario, a reduction in greenhouse gas emissions of 9.03% and 11.82% respectively by 2025 and 2030 compared to the baseline situation; and according to the conditional scenario 14.64% and 24.28% by 2025 and 2030 compared to the baseline situation”	13,444.3	0.025%
	Chad	2020	“The target is to reduce GHG emissions by 19.3% in 2030 compared to the Baseline Case. The unconditional scenario based on the country's own resources will lead to a 0.5% reduction in emissions compared to the reference scenario in 2030. The implementation of the conditional scenario, based on international support, would allow a total reduction in emissions of 19.3%”	101,173.8	0.190%
	Republic of Congo	2020	“In terms of mitigation, the Republic of Congo remains a net carbon sink and aims to strengthen this positive contribution to the global climate by 2035. The unconditional scenario takes into account the current low-carbon measures in the sectors and those for which the country does not expect external support for their implementation. Thanks to these efforts, the country is gradually strengthening its performance to −21,717 ktCO_2_eq in 2030, an improvement of 7.8% compared to the BAU scenario of the same year. By 2035, removals will reach-−21,932 ktCO_2_eq, a 22% improvement compared to the BAU, reflecting the consolidation of the country's role as a carbon sink through the programmes already operational. By adding additional measures that require external support (combined Unconditional + Conditional scenario), net removals increase from −22,033 ktCO_2_eq in 2025 to −24,254 ktCO_2_eq in 2030, an improvement of 20.4% compared to the BAU. By 2035, removals will reach −26,569 ktCO_2_eq, an increase of 47.8% compared to the BAU”	24,623.0	0.046%
			MtCO2e compared to the reference scenario, i.e., a reduction of around 20.15% over the period 2021–2030”		
	Democratic Republic of Congo	2020	“The combined unconditional and conditional contribution is…a 21% reduction in total GHG emissions compared to the BAU in 2030 (of which 19% conditional and 2% unconditional); this equates to an estimated mitigation level of up to 650 MtCO2e by 2030”	58,491.7	0.110%
	Cote d'Ivoire	2025	“…NDC 3.0 sets an ambitious target of reducing emissions by 33.07% by 2035 compared to the BAU scenario, by mobilizing a portfolio of differentiated sectoral measures: unconditional measures: constitute an implementation commitment, allowing emissions to be reduced to 104,659 GgCO2eq, i.e., a decrease of 33.07% compared to the BAU scenario; Conditional measures: depending on increased international support (finance, technology transfer, capacity building), they would offer a more ambitious potential, limiting emissions to 40,199 GgCO2 eq, a reduction of 74.29%”	38,026.7	0.071%
	Equatorial Guinea	2020	“Equatorial Guinea's ambition in this CDN update is to reduce emissions by 35% by 2030, with the goal of reaching 50% by 2050”	8,138.7	0.015%
	Gabon	2025	“Maintain and strengthen Gabon's carbon neutrality beyond 2050”	21,782.0	0.041%
	Gambia	2020	“The mitigation measures described in the NDC2 will allow the country to reduce its GHG emissions by 49.7 percent compared to the expected 2030 baseline. In absolute figures, the mitigation measures will reduce The Gambia's GHG emissions by 3,290 GgCO2e”	1,859.5	0.003%
	Ghana	2020	“ For the 34 mitigation measures, Ghana aims to implement nine unconditional programmes of action that would result in 8.5 MtCO2e GHG reductions by 2025 and a further 24.6 MtCO2e by 2030 compared to the 2020–2030 cumulative emissions in a baseline scenario. Ghana can also adopt additional 25 conditional programmes of action that have the potential to achieve 16.7 MtCO2e by 2025 and 39.4 MtCO2e by 2030 if financial support from the international and private sector is made available to cover the full cost for implementation”	48,093.9	0.090%
	Guinea	2025	“Unconditional target: 9.7% reduction in non-AFOLU GHG emissions by 2035 (target emissions: 14,035 ktCO_2_e) Conditional objective: 20% reduction in non-AFOLU GHGs by 2035 (target emissions: 12,434 ktCO_2_e) All sectors: Unconditional target: 17% reduction in GHG emissions with AFOLU by 2035 (target emissions: 83,052 ktCO_2_e) Conditional target: 49% reduction in GHGs with AFOLU by 2035 (target emissions: 51,032 ktCO_2_e)”	29,375.9	0.055%
	Guinea-Bissau	2020	“The Guinea-Bissau contribution aims to reduce GHG emissions by 30% in 2030 compared to the baseline scenario. The unconditional contribution based on the country's own resources would lead to a 10% drop in GHG emissions by 2030 compared to the reference scenario. The conditional contribution, based on international support, would allow an additional reduction in emissions of 20%”	3,188.6	0.006%
	Liberia	2025	”Liberia reaffirms its ambitious commitment to reduce economy-wide greenhouse gas emissions by 64% by 2035. However, following a change in the base year from 2015 to 2022—where the calculation of emissions rose from 5,695 GgCO_2_e to 12,471 GgCO_2_e due to improved data and methodologies—this reflects a three-fold increase in GHG emissions. As a result, Liberia updated its Business-As-Usual (BAU) and mitigation scenarios. The revised 2035 targets are: an unconditional reduction of 5,551 GgCO_2_e (10%) and a conditional reduction of 29,974 GgCO_2_e (54%), contingent on international support”	4,459.9	0.008%
			MtCO2e compared to the reference scenario, i.e., a reduction of around 20.15% over the period 2021–2030”		
	Mali	2020	“Mali commits to reduce emissions by 31% for energy, 25% for agriculture, 39% for land use and forestry, and 31% for waste sectors by 2030 compared to BAU”	48,008.5	0.090%
	Mauritania	2025	“Mauritania commits to a reduction of 75.8% (i.e., 17,433.84 Gg CO2eq) in 2035 compared to the reference level (BAU), including an unconditional reduction of 8.1% (i.e., 1,873 Gg CO2eq) and a conditional reduction of 67.7% (i.e., 15,561 Gg CO2eq)”	17,508.6	0.033%
	Niger	2020	“AFAT sector: Unconditional Reductions: 4.50% (BAU 2025) and 12.57% (BAU 2030) and Conditional Reductions: 14.60% (BAU-2025) and 22.75% (BAU 2030). Energy sector: Unconditional Reduction: 11.20% (BAU-2025) and 10.60% (BAU-2030) and Conditional Reductions: 48% (BAU-2025) and 45% (BAU-2030)”	41,146.1	0.077%
	Nigeria	2025	“The Federal Republic of Nigeria sets an ambitious target to achieve an absolute emissions reduction of 184.9 Mt CO2e in 2035 from the emissions of 573.5 Mt CO2e in 2018, which represents a 32.2% reduction”	350,555.4	0.659%
	São Tome and Principe	2025	“35.4% reduction in GHG emissions in 2035, relative to the BAU scenario, excluding the Land-Use, Land-Use Change and Forestry (LULUCF) sector”	245.5	0.000%
	Senegal	2020	“Senegal commits to reduce GHG emissions by 5% and 7% (unconditional) and 23.7% and 29.5% (conditional) compared to BAU levels in 2025 and 2030 respectively”	31,720.6	0.060%
	Sierra Leone	2025	“Reduce GHG emissions from the waste sector by 52.5% below BAU levels by 2035; Reduce GHG emissions from the transport sector by 16.2% below BAU levels by 2035; Reduce GHG emissions from the energy sector by 41.6% below BAU levels by 2035; Reduce GHG emissions from the AFOLU sector by 33.6% below BAU levels by 2035”	7,928.5	0.015%
	Togo	2020	“Unconditional contribution: The results of the analysis of sectoral reductions indicate that Togo can commit to an unconditional contribution to reduce its greenhouse gas (GHG) emissions by 20.51% by 2030, or 6,236.02 GgCO2eq. Conditional contribution: in the proposed approach for the mitigation scenario, the Togolese State undertakes, if it receives the required support, to achieve a further 30.06% reduction in GHG emissions compared to the reference scenario by 2030, i.e., 9,305.59 GgCO2eq. without compromising its food self-sufficiency policy by proceeding in such a way as not to compromise its sustainable development”	10,844.3	0.020%
**Total**	**All Countries**			**3,338,078.6**	**6.274%**

Targeted emission goals are taken from countries' most recent Nationally Determined Contributions, as quoted in the Climate Watch tracker (World Resources Institute 2025) (135). Data on 2024 emissions is taken from the European Union's Emissions Database for Global Atmospheric Research (EDGAR 2025) (136).

An additional ethical lens is offered by Ubuntu, the African philosophical tradition grounded in relational humanity, reciprocity, dignity, and interdependence. Ubuntu emphasizes that individual wellbeing is inseparable from collective wellbeing and that justice requires solidarity with those most exposed to harm. Applied to climate governance, Ubuntu challenges the market-based responses of developed countries with an alternative vision that prioritizes community resilience, shared management of natural resources, intergenerational responsibility, and protection of vulnerable groups. It highlights why externally imposed policies that overlook local participation, traditional knowledge, and social cohesion fail to achieve sustained uptake (16).

**Figure 1:**
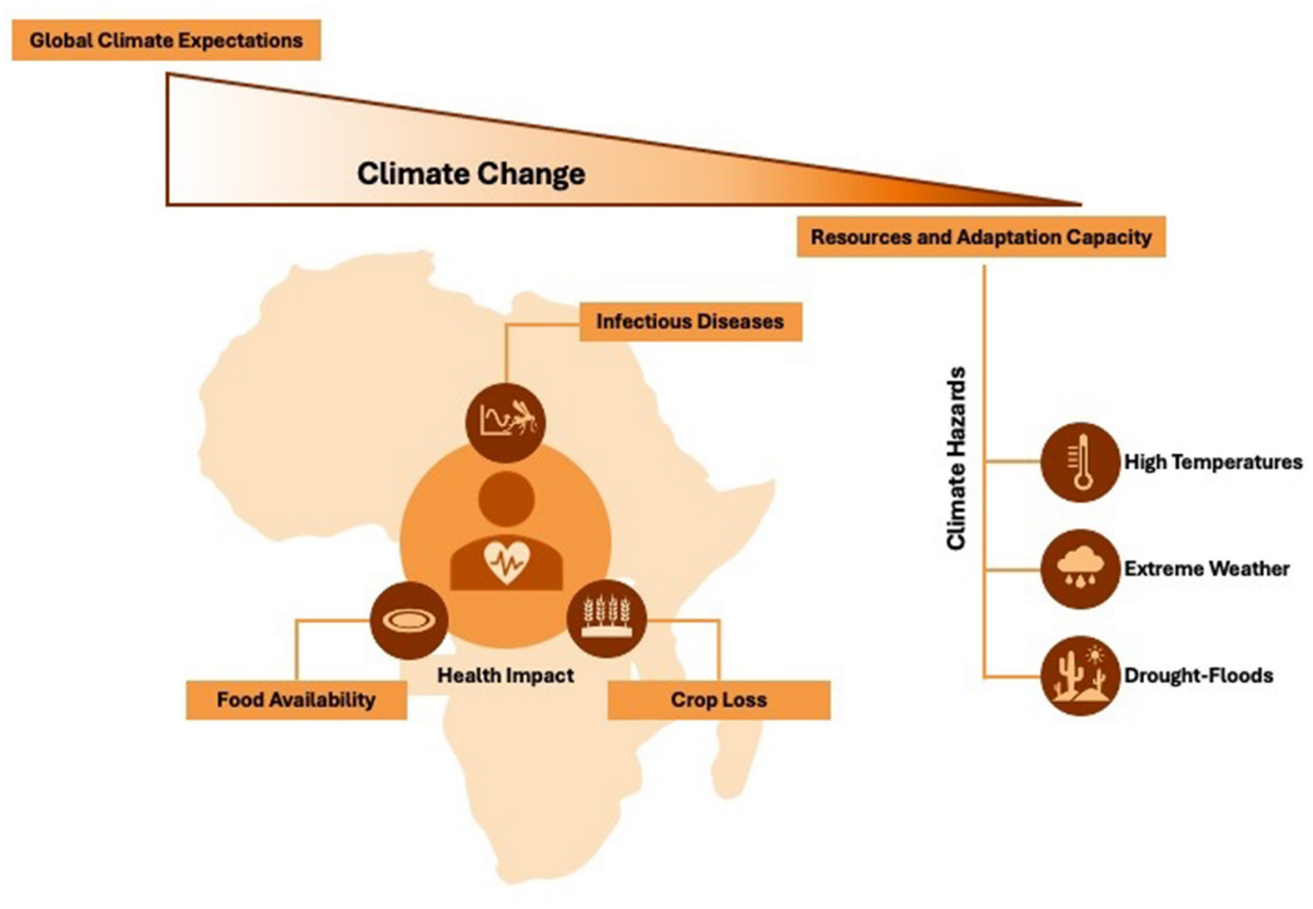
Schematic representation of global expectations vs. resource availabilities. The figure illustrates the discrepancy in the contribution expected from Africa in the fight against climate change in relation to the many challenges that the continent faces regarding insufficient financial support and human capital.

In this review, we highlight the structural limitations preventing Africa from responding effectively to climate change. This article was conducted as a narrative review with integrative policy analysis, chosen to synthesize multidisciplinary evidence spanning climate science, public health, environmental governance, development economics, and ethics. A narrative approach, emphasizing emerging scientific literature from the last decade, was considered most appropriate because the research question addresses complex structural relationships and policy translation challenges. By centering on global inequities and policy designs that insufficiently account for African socio-economic realities (3, 10, 17), this reflection is a wake-up call to action. It argues for a collective shift toward strategies that are context-sensitive, health-centered, and firmly grounded in principles of climate and health justice (Figure 1).

Such strategies should be co-designed with African institutions and communities, implemented according to countries' capacity and needs, and evaluated through quantifiable improvements in health, livelihoods, and resilience rather than on the basis of compliance alone. A central policy implication of this review is that international climate finance and adaptation frameworks should explicitly prioritize health systems resilience in Africa, including primary care infrastructure, climate-health surveillance, water and sanitation systems, and community-based preparedness, as core indicators of successful climate action rather than treating health as a secondary co-benefit. Only by aligning global ambition with local feasibility can global climate policy can avoid reproducing historical inequities and instead achieve the goals of climate justice and health equity across Africa.

### Regional heterogeneity across Africa

Strengthening climate adaptation, defined by the United Nations Framework Convention on Climate Change (UNFCCC) as actions intended to “moderate potential damages or to benefit from opportunities associated with climate change” (18), is the most immediate and critical priority for protecting health in Africa (10). Despite this urgency, only a minority of total climate finance is directed toward adaptation strategies as compared to mitigation strategies (12). This imbalance reflects a global preference for emissions-reduction investments that generate measurable carbon outcomes, whereas adaptation benefits are often diffuse, locally specific, and harder to quantify despite their immediate relevance for human health. Even conservation measures implemented across the continent often replicate models developed in high-income countries, emphasizing protected areas while insufficiently integrating local livelihoods. This undermines adaptation efforts and, in some contexts, exacerbates poverty and pre-existing social inequities (19, 20). Where conservation restricts land, water, or fishing access without compensation, communities may bear disproportionate costs while receiving limited resilience benefits.

Importantly, these climate impacts are not experienced uniformly. Their intensity and consequences are mediated by pre-existing differences in income distribution, infrastructure coverage, livelihood dependence on climate-sensitive sectors, governance capacity, and access to health and social protection systems. Climate change amplifies uneven development, while deepening territorial and social disparities between and within African countries. Marginalized populations often experience higher levels of risk from environmental exposures, while facing lower capacity to respond and adapt (21, 22). Social determinants such as financial savings, secure housing, irrigation, insurance, mobility, or access to quality health and healthcare services shape the population-level effects of droughts, floods, or extreme heat events. Across African nations, the common denominators shaping such outcomes include the quality of governance institutions, the presence or absence of resilient infrastructure, and the degree of socio-economic inequality. Identical climate stressors can lead to disparate health outcomes across countries facing differing levels of food insecurity, displacement, excess morbidity, and long-term developmental losses (12, 23, 24).

Across the African continent, regional heterogeneity characterizes climate change interactions with respect to governance capacity, infrastructure, economic structure, and social inequalities. Representative examples have been selected to illustrate how climate impacts are mediated by distinct regional political economies and developmental trajectories. This analysis, although limited in its ability to account for the diversity of all national and sub-national contexts, reemphasizes that adaptation strategies cannot be approached through uniform policy frameworks. The following section aims to illustrate this sub-regional variation as context to ground the discussion of specific climate-related health interactions that follows.

Compared to many other African nations, Northern African countries display stronger administrative capacities. This can be attributed, to an extent, to their partial regulatory alignment with the climate and environmental frameworks of the European Union, which facilitates large-scale investments and positions this region as a continental leader in mitigation-oriented infrastructure (25). However, this advantage does not translate into reduced climate vulnerability. In Morocco, for instance, water scarcity remains a major challenge and, considering intermediate warming scenarios, cereal yield is estimated to decline by approximately 10%−20% by 2050 (26, 27). These projections are expected to increase food price volatility and nutritional risk, exacerbating the inequalities often observed between rural and urban populations. Similar vulnerability is also expected in Egypt, where heat and flood stress may reduce fiscal buffers and increase sensitivity to food and water price fluctuations (28, 29). Such examples illustrate that stronger state capacity can reduce implementation barriers, but cannot fully offset structural exposure where water scarcity, import dependence, and socio-spatial inequality remain pronounced (26).

In Western and Central Africa, the agricultural sector encompasses approximately 40%−60% of the labor force (30). Over 90% of crops are rain-fed, making local livelihoods highly sensitive to climate change. This vulnerability is markedly pronounced across the Sahel region, given its reliance on subsistence agriculture, pastoralism, and agropastoral mobility systems. Those activities are continuously disrupted by recurrent droughts, land degradation, desertification, and rainfall variability (31, 32). Rapid population growth, fiscal constraints, governance fragility, and territorial insecurity limit the ability of institutions in the region to implement long-term adaptation policies. Those challenges also reduce the operational reach of agricultural extension programs and climate surveillance systems, especially in rural and border regions, rendering climate adaptation reactive rather than structurally integrated into planning processes (33). Additionally, interactions between rapid coastal urbanization, flooding, and governance pressures are prominent across the region. For example, accelerated coastal erosion in Senegal repeatedly threatens fishing communities and displaces local residents (34, 35). Small Island States, such as Cabo Verde and São Tomé and Príncipe, also exhibit pronounced climate sensitivity due to their unique characteristics of geographic isolation, narrow economic diversification, dependence on marine resources, and limited adaptive fiscal space (36). In the face of persistent water scarcity and land aridity, fisheries have remained central to the livelihoods of these nations. However, this increased dependence on marine ecosystems is now compromised by ocean warming/acidification, overfishing, and plastic pollution, while governance fragmentation across rapidly growing coastal cities further complicates drainage management, land-use regulation, and coordinated disaster response (37). The diversity of climate risk profiles within Western and Central Africa further reinforces the need for differentiated adaptation frameworks which integrate ecological conditions, livelihood systems, state capacity, and security dynamics.

Increased drought frequency also affects East Africa, where climate-sensitive pastoralist and agropastoralist systems are predominant (38). Malnutrition rates in Kenya and Ethiopia have increased in recent years, likely due to severe droughts affecting millions of people and causing significant livestock losses. Crop suitability is also being altered by rising temperatures, which compromises long-term food security (39). These pressures are particularly severe in fragile and conflict-affected settings, such as South Sudan and Chad, where livelihoods depend on rain-fed agriculture, pastoral mobility, fisheries, and livestock systems that are highly sensitive to hydrological variability. In South Sudan, the challenges posed by recurrent flooding and drought cycles, combined with political instability, population displacement, and limited state infrastructure, hinder the implementation of long-term climate adaptation and health policies (40). Similarly, in Chad, progressive desertification, declining pasture availability, and water scarcity intensify competition over land and water resources within pastoralist and agricultural communities. These environmental pressures, as linked to territorial insecurity, weak institutional presence, and fiscal limitations, reduce the capacity to operationalize climate adaptation frameworks beyond emergency response interventions (40). Across the Horn of Africa and neighboring Sahelian-transition zones, climate change increasingly acts as a threat multiplier, exacerbating pre-existing fragilities linked to displacement, resource competition, and governance systems (41). In those contexts, adaptation policies are often implemented under conditions of persistent humanitarian pressure, where immediate survival needs compete with long-term resilience planning.

Intensified climate events are also felt in Southern African countries, where social inequalities amplify the relationship between exposure and adaptation capacity (23, 42). Extreme weather events tend to generate the greatest losses among households with precarious housing, informal employment, and limited financial savings, while recovery is faster among wealthier groups with insurance, mobility, and stronger political representation. Compared with other African regions, Southern Africa combines relatively stronger infrastructure corridors and financial sectors (43). However, persisting vulnerability in this region can be witnessed in countries such as Mozambique, which is continuously affected by high cyclone frequencies and floods, which then cause major socio-economic disruptions. Across the region, climate change is not only an environmental threat, but a structural force that reproduces inequality.

This brief comparison indicates that no single narrative on climate challenges in Africa is sufficient to capture regional complexities and nuances. North Africa is more constrained by water scarcity and price transmission; Western and Central Africa by rainfall dependence, coastal exposure, and urban governance pressures; East Africa by drought-driven livelihood erosion and food crises; and Southern Africa by environmental extremes interacting with spatial inequality. However, across all regions, climate vulnerability goes beyond environmental exposure, as distinct deficits in infrastructure, uneven governance capacity, and entrenched socio-economic disparities remain the principal channels through which climate hazards become health and developmental crises. Equity-oriented adaptation must, therefore, be differentiated by region while addressing shared structural challenges visible throughout the continent.

### Health system, climate shocks and financing gaps

Across regions, inadequate investment in urban drainage, waste management, and resilient infrastructure exacerbates the health impacts of extreme climate events. By compromising food availability and increasing the risk of infection outbreaks, these vulnerabilities sustain the pressure on health systems that are already fragile. However, the magnitude of these effects differs substantially across African regions according to governance quality, infrastructure coverage, fiscal capacity, and underlying socio-economic inequality. In countries where urban planning systems, service delivery institutions, and public health networks are more effectively implemented and integrated, such systems serve as a buffer against climate shocks and their disruptions in the form of displacement, food insecurity, and excess morbidity (12, 23, 24). Regional patterns, as outlined in the previous section, further illustrate this divergence.

Many African countries lack comprehensive early warning systems, crop insurances, and other fiscal strategies to mitigate extreme climate events (44). These interventions force government to routinely analyze and divert public resources toward the recurring implementation of emergency health responses. Where contingency financing, social protection systems, and sovereign risk-transfer instruments are limited, governments often reallocate budgets from long-term priorities such as primary health care, infrastructure maintenance, and education toward short-term crisis management. Over time, this repeated fiscal diversion reduces development gains and weakens adaptive capacity (45).

Furthermore, access to climate finance mechanisms, many administered by international entities, is often conditional upon complex eligibility and reporting requirements that often exceed national administrative capacities. Application procedures frequently require detailed vulnerability assessments, fiduciary safeguards, procurement systems, monitoring frameworks, and co-financing commitments that are easier to meet in countries with larger bureaucracies and readily-available technical pools. For many lower-capacity states, scarce administrative personnel must be diverted from routine governance functions to navigate fragmented funding architectures, multiple donor templates, and recurrent compliance exercises (46, 47). As a result, available funding is often requested through loans rather than grants, which ultimately increases public debt. This pattern is especially problematic in the most climate-vulnerable countries where fiscal space is already constrained by external debt servicing, import dependence, and recurrent disaster expenditure. This dynamic generates a self-reinforcing cycle of indebtedness that limits sustained investments in foundational infrastructures essential for climate resilience and health system strengthening (12, 48, 49). Rising debt service can limit public expenditure on primary care, water systems, sanitation, agricultural extension, social protection, and maintenance of protective infrastructure, thereby increasing future vulnerability and creating additional demand for emergency finance after subsequent shocks.

Climate finance can therefore become reactive rather than transformative when not structured on concessional and predictable terms. Further challenges lie in the predominance of project-based financing over long-term systems financing. Short funding cycles often privilege visible infrastructure or pilot programs, while underfunding recurring costs such as workforce salaries, surveillance operations, maintenance of drainage systems, laboratory networks, and community outreach. Yet these recurring functions are precisely what determine whether adaptation investments remain operational during crises. Fragmented financing can also generate parallel reporting systems and donor dependence, weakening national ownership and planning coherence.

These constraints have direct public health consequences. For example, post-flood conditions are often associated with infections (which are frequently lethal), nutritional deficits, and psychological distress. Repeated flood and drought emergencies are additionally linked to interruptions in vaccination, maternal care, chronic disease management, and schooling, demonstrating how climate-sensitive infrastructure failures can generate long-term human capital losses beyond immediate mortality and morbidity (12).

From a policy perspective, reform of climate finance mechanisms is essential. Priority actions include increasing the share of grant-based adaptation finance; simplifying application and reporting procedures; expanding direct access windows for national and subnational institutions; aligning disbursement criteria with vulnerability rather than administrative sophistication; and financing recurrent system costs alongside capital investments. Debt swaps for climate and health resilience, contingency financing for shocks, and predictable multi-year support for primary health systems could further reduce the cycle through which disasters deepen indebtedness and indebtedness deepens disaster risk (50, 51).

Ultimately, climate finance should be evaluated on the basis of whether it measurably reduces exposure, strengthens public systems, and protects health in the countries facing the greatest climate burdens. Without such recalibration, current financing architectures risk reproducing the very inequalities they are intended to address. Strengthening climate resilience requires not only emergency preparedness, but sustained investment in inclusive institutions, risk-informed infrastructure, and equity-oriented public finance capable of reducing structural vulnerability across regions.

### Impacts of climate change on food security and nutrition in Africa

In recent decades, our planet has entered a distinct climatic epoch, characterized by the accelerating impact of human activity on planetary boundaries. Although temperature shifts are not uniform, the World Meteorological Organization (WMO) has identified the last decade as the warmest registered in Africa (52). Recurrent multi-season droughts and flood episodes have compromised agricultural productivity, disrupted food and water systems, and led to severe consequences for populations that already face poverty and limited access to health services (4, 12). In recent years, torrential rainfalls have affected millions of people, resulting in widespread population displacement and livelihood loss (52). These impacts are not evenly distributed across the continent. Their severity depends on the interaction between climatic exposure and aforementioned structural determinants such as governance capacity, infrastructure quality, dependence on climate-sensitive livelihoods, and levels of social inequality.

Regional contrasts are particularly instructive. North Africa has experienced rapid warming, chronic water scarcity, and heightened food import vulnerability, meaning that climate impacts are often transmitted through water stress, heat exposure, and price inflation rather than crop failure alone. West Africa is characterized by high rainfall variability, coastal flooding, and strong dependence on rain-fed agriculture, making both rural livelihoods and rapidly expanding cities acutely sensitive to seasonal extremes. East Africa has faced repeated multi-season droughts that have undermined pastoral systems, livestock assets, and nutrition security, while Southern Africa increasingly confronts compound risks from heatwaves, drought, and cyclones interacting with highly unequal social structures (23, 24). These comparisons indicate that climate change in Africa is not a single continental phenomenon, but a set of regionally differentiated stress pathways which manifest in distinct health and developmental outcomes.

Indicators derived from agricultural and food systems provide a particularly valuable lens for assessing climate change, integrating changes in weather parameters with their downstream socio-economic consequences (53). In the last 5 years, agricultural production decreased below average in several regions of Eastern Africa. Maize and wheat crops were particularly affected, as influenced by heat-driven evapotranspiration and stress during critical growth periods (54, 55). Each additional degree in temperature increase is associated with an average reduction in maize yields of approximately 7.4% (56). In locations where rain-fed agriculture predominates, climate pressures translate into food insecurity, which is further aggravated by the lack of mechanisms for market stabilization and mitigating high price volatility (57). This relationship is especially pronounced in West and East Africa, where millions of households depend directly on smallholder or pastoral production and possess limited savings or irrigation access. By contrast, regions with more diversified economies or larger fiscal buffers may absorb agricultural losses through imports and subsidies, although often at substantial budgetary cost. Thus, governance and market capacity strongly mediate whether production shortfalls become nutritional crises.

Infrastructure deficits further amplify agricultural risks. Inadequate roads in rural areas, storage facilities, electrification, and cold chains increase post-harvest losses, constrain internal food redistribution, and intensify local scarcity during drought or flood years (58–60). In fragile settings, climatic disruption enhances dietary deterioration, which in turn increased pressure on health systems through undernutrition and infection susceptibility.

Although the main discussions on climate and nutrition prioritize agricultural production, aquatic food systems provide complementary insights, demonstrating that climate change is not confined to atmospheric processes (61). Ocean-surface temperatures surrounding the African continent have reached their highest records in the last decade, particularly in the Atlantic and Mediterranean-adjacent regions (62). The increased coastal exposure of these locations enhances their risk of flooding and shoreline erosion, thus threatening food security and the livelihoods of fishery-dependent populations and coastal ecosystems. These considerations are especially relevant for countries that rely on marine resources but do not possess sufficient cold-chain infrastructures to maintain fish at low temperature from harvest and provide a controlled supply to consumers. The socio-economic implications of these challenges vary by region. In North and West Africa, marine warming and coastal erosion threaten artisanal fisheries, port infrastructure, and densely populated coastal settlements. In Small Island Developing States such as Cabo Verde, fisheries disruptions may have disproportionate consequences due to the strong reliance on marine resources for employment, protein intake, and foreign exchange earnings. In Southern Africa, ocean warming may alter species distribution and catch composition, affecting both industrial and small-scale fisheries. Where governance of marine resources is weak or enforcement capacity limited, climate stress can combine with overfishing and pollution to accelerate ecosystem decline (63).

Taken together, terrestrial and aquatic indicators show that climate change in Africa is best understood through a systems lens. Environmental stress interacts with infrastructure gaps, unequal development, and governance capacity to determine whether hazards remain manageable or escalate into widespread food, health, and livelihood crises. Adaptation strategies need to move beyond hazard monitoring alone and strengthen the institutions, markets, and public services that convert exposure into resilience.

### Nutritional outcomes as indicators of climate adaptation

Nutritional outcomes provide measurable evidence of climate-driven health impacts. A multi-country analysis across 30 sub-Saharan African countries demonstrated that higher ambient temperatures were consistently associated with poorer child nutritional outcomes, affecting both acute weight-based indicators and chronic growth parameters (64). Complementary work on food availability, infection exposure and healthcare access highlight undernutrition as one of the most climate-sensitive health outcomes in low-resource countries (65, 66). Viewed through a One Health lens, these nutritional outcomes cannot be separated from animal health, ecosystem integrity, food safety, and the stability of agricultural and livestock systems. In North and sub-Saharan Africa, where livelihoods frequently depend on close human–animal–environment interactions, climate change affects nutrition not only through crop failure, but also through livestock morbidity, reduced milk and meat availability, zoonotic disease risks, water contamination, and degradation of ecosystems that sustain food production (67–69).

Even small changes in harvests and purchasing capacity can rapidly compromise dietary adequacy, especially when compounded upon pre-existing nutritional deficiencies. Meanwhile, infectious disease burdens rise during extreme climate events, creating synergistic risks for child and maternal health (70). These interactions are particularly important in pastoralist, agropastoralist, and mixed farming systems, where droughts and floods also intensify household exposure to food-borne, water-borne, and zoonotic pathogens. The recognition that environmental stressors can be nutritionally catastrophic, especially due to supply chain constraints, has renewed governmental attention to diet quality and micronutrient deficiencies (71). Climate stress reduces dietary diversity by limiting the availability and affordability of nutrient-rich foods, such as legumes, fruits, vegetables, and animal-based products, while increasing reliance on unhealthy processed products (72). This shift has profound implications for immune function, cognitive development, and maternal–child health, as deficiencies in iron, zinc and vitamin A are strongly associated with increased infection susceptibility, adverse pregnancy outcomes, and impaired early neurodevelopment (73–79). The reduction in animal-source foods is especially consequential because these foods are dense sources of bioavailable micronutrients and high-quality protein. However, their availability is increasingly threatened by heat stress, pasture degradation, water scarcity, animal disease, and disrupted veterinary services.

In parallel, evidence accumulated over the past decade indicates that changing atmospheric composition can directly affect the nutritional quality of cultivated products. Elevated concentrations of carbon dioxide reduce zinc and iron content in C3 grains and legumes, indicating that climate change may worsen existing micronutrient deficiencies observed in many populations (17, 80). Although this phenomenon has global relevance, it is particularly concerning for Africa, where diets rely heavily on staple grains and legumes. A One Health approach further expands this concern by linking nutritional quality to food safety, in terms of increased contamination risks by mycotoxins, chemical pollutants, unsafe water, and pathogen transmission across human, animal, and environmental interfaces (81).

**Figure 2:**
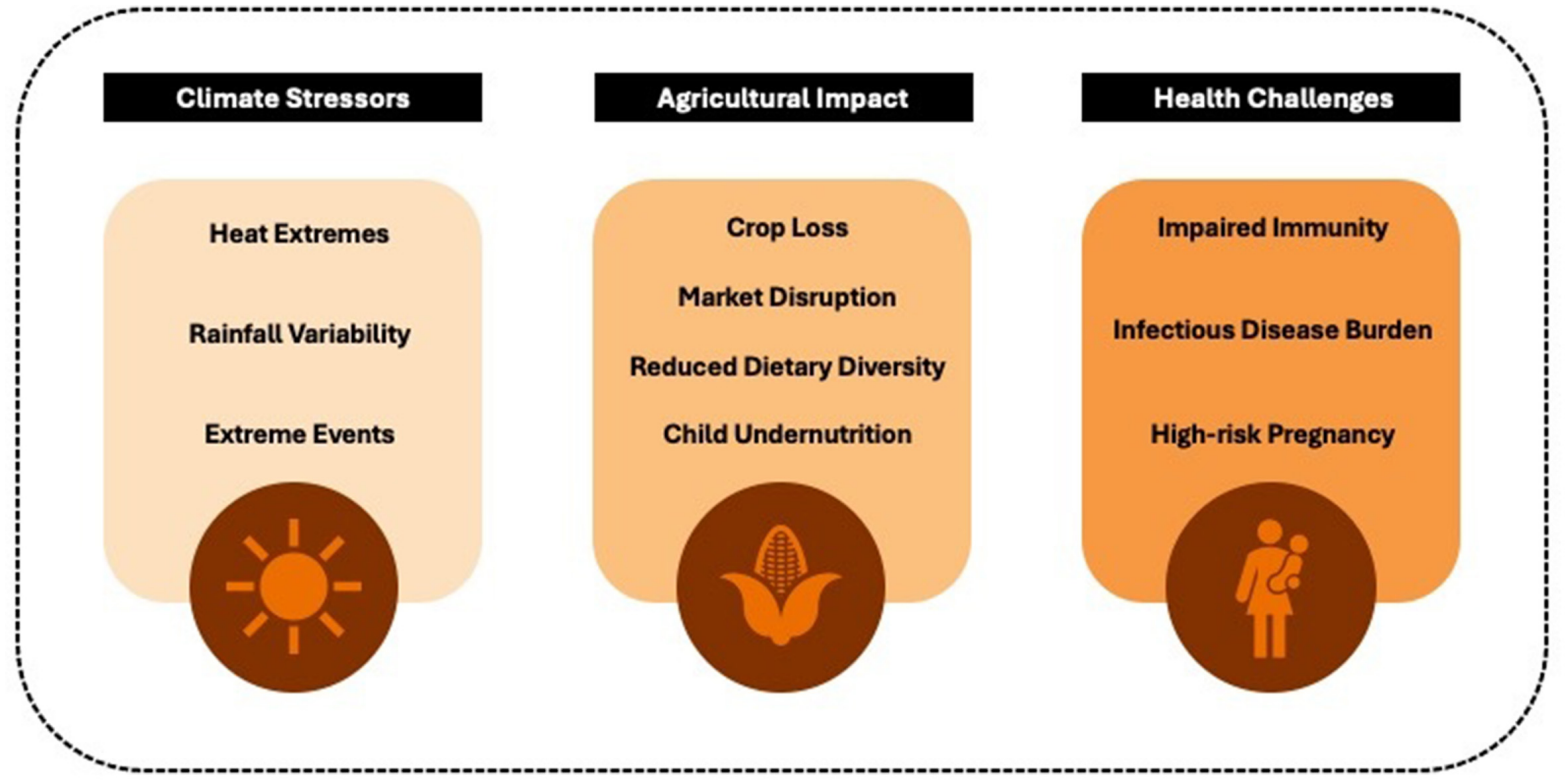
Simplified schematic representation of the main considerations for climate adaptation. The figure illustrates typical climate stressors, as well as their agricultural and health impacts.

Hence, the health consequences of climate change extend beyond malnutrition, also posing a significant threat to nutrient density and biological integrity (67). Nutritional stress increases the risk and severity of infectious diseases, amplifying the economic and health-related burden of epidemic outbreaks. This notion underscores the need for climate adaptation strategies, such as nutrition-sensitive agriculture, resilient food storage and cold chain systems, to stabilize agricultural markets and protect population health (64, 82) (Figure 2).

While still emerging in the scientific literature, it is also important to understand how climate-sensitive food insecurity interacts with other chronic health challenges, including diarrheal disease, antimicrobial resistance pressures, and mental health distress (83). This latter, especially, is increasingly recognized as an important but still under-characterized consequence of climate change. Mental health impacts are frequently associated with livelihood loss, forced displacement, food insecurity, reduced access to healthcare and education, worsening socio-economic marginalization, and increased gender-based violence and exploitation in contexts of heightened vulnerability (84). These interconnected consequences are particularly severe in settings where health (care) systems remain fragile (85). Hence, future work is needed to develop culturally appropriate and locally grounded interventions, addressing this topic, especially, at the community level.

### Climate change and vector-borne diseases in Africa

Malnutrition and nutrient deficiencies additionally intersect with climate-driven ecological changes and shape vector-borne disease risk across Africa (4). These interactions arise due to the influence of environmental alterations on mosquito ecology. By expanding the geographical range and seasonal persistence of transmission, climate change has been shown to significantly impact the epidemiology of mosquito-borne and other vector-borne infections (86, 87). Recent evidence further reinforces that these dynamics should be interpreted through an integrated environmental health perspective, in which climate change, urbanization, biodiversity disruption, pollution, food insecurity, and public infrastructure are viewed as converging determinants of infectious disease emergence and transmission. In many African settings, rapid environmental transformation is reshaping the interfaces between humans, vectors, animals, and ecosystems, thereby increasing exposure to multiple climate-sensitive health threats simultaneously (68).

Thermal and hydrological conditions affect mosquito survival, proliferation, biting rates, and the speed at which pathogens develop within these vectors, thereby modulating their transmission potential (88). Temperature is a dominant driver of these processes, defining vectorial capacity and transmission intensity (89, 90). At higher temperatures, transmission risk increases, reaching an optimal threshold beyond which mosquito survival declines and transmission efficiency is reduced (91). This relationship indicates that global warming can increase malaria burden in countries and areas that have so far experienced low incidence (92, 93). These shifts are particularly concerning in populations already affected by malnutrition and chronic health stress, as nutritional deficiencies may compromise immune function and increase susceptibility to severe infectious outcomes. Consequently, climate change simultaneously modifies ecological transmission dynamics and population health vulnerability (68).

**Figure 3:**
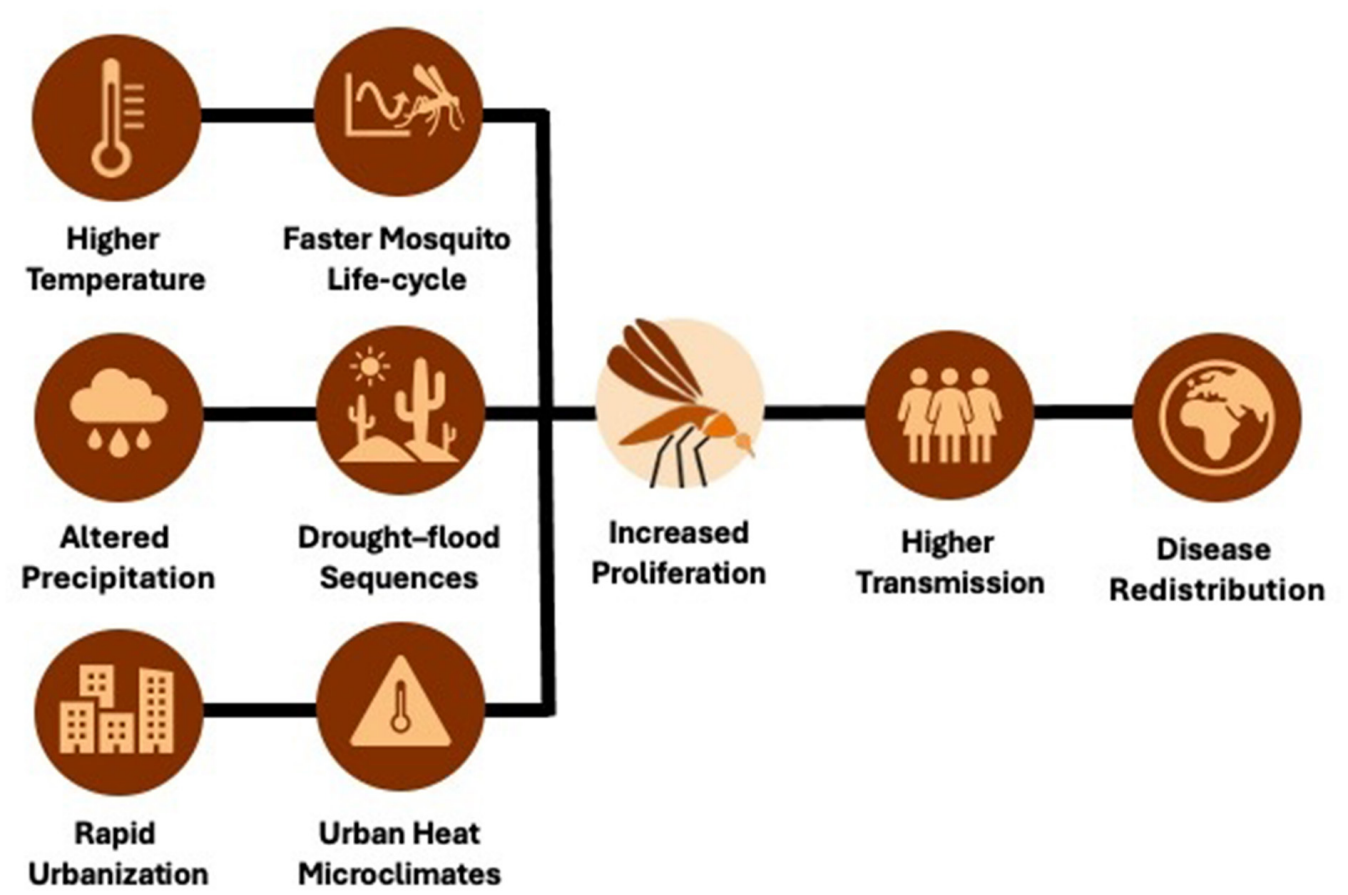
Schematic representation of the main contributing factors to an increase in vector-borne disease transmission. The figure illustrates the impact of different variables on mosquito proliferation. The adaptability of these vectors to different climate conditions enhances transmission rates and influences their global distribution.

Three major ecological mechanisms have been identified as driving shifts in malaria incidence. First, higher temperatures shorten the length of the mosquito life cycle, which increases both feeding frequency and, consequently, the number of potentially infectious bites per person (88, 90). Second, altered precipitation patterns, characterized by more frequent drought–flood sequences and episodes of intense rainfalls, alter the availability and persistence of larval habitats. Ultimately, this favors mosquito breeding (94, 95). These dynamics are well described for *Anopheles* mosquitos, which transmit malaria parasites (96). Additionally, drought-related practices of household water storage can expand breeding habitats for *Aedes* species, which spread viral diseases such as yellow fever, dengue, Zika and Chikungunya (97–99). Environmental degradation associated with poor urban planning, deforestation, waste accumulation, and water contamination may further intensify these transmission dynamics by creating highly suitable ecological niches for vectors. Plastic waste accumulation, for example, contributes to standing-water habitats favorable to mosquito breeding, particularly in densely populated informal settlements where drainage systems are insufficient or absent (68). Third, rapid urbanization across many African cities contributes to the formation of urban heat microclimates that allow mosquitoes and pathogens to persist longer; this results in sustained transmissions across seasons (100). The absence of consistent drainage, solid waste management, and reliable tap water in many African cities further amplify transmission risk (101, 102) (Figure 3).

These conditions illustrate how vector-borne disease risk in Africa is increasingly embedded within broader environmental and developmental transitions. Unplanned urban expansion, informal settlements, air and water pollution, biodiversity loss, and inadequate infrastructure interact with climate variability to generate environments where infectious diseases can spread more efficiently. This reinforces the need for integrated climate–health governance approaches guided by One Health and Planetary Health frameworks, in order to prioritize and protect human wellbeing in the face of global challenges.

### Vulnerabilities and equitable climate-informed vector control strategies

As demonstrated by the examples in the previous three sections, the health consequences of climate–vector dynamics in Africa are both acute and direct, as well as chronic and systemic. Changing climates increase the likelihood of more frequent outbreaks, sustained transmission, and the emergence of vector-borne diseases in regions with limited population immunity and lower clinical suspicion (92, 103, 104). These diseases also exacerbate health system vulnerabilities by increasing the demand for diagnostics, treatment, and outbreak response. Such emergencies also divert scarce resources away from routine and preventive care (3, 104). Across health indicators, social inequalities increase disease susceptibility among marginalized groups, including children, pregnant women, displaced populations and residents of informal settlements. These individuals experience higher exposure and worse health outcomes due to structural deficits in housing, water access, and health service coverage (105–107).

More broadly, vector-borne disease risk is shaped by the same socio-economic inequalities that structure climate vulnerability across Africa. Poverty, insecure livelihoods, informal urbanization, weak infrastructure, and uneven territorial development often concentrate vulnerable populations in environments where mosquito breeding is more likely and protective services are less available. Households lacking screened housing, reliable piped water, sanitation, drainage, or waste collection are more frequently exposed to vector contact, while delayed access to diagnosis and treatment increases the probability of severe outcomes. In many settings, climate change magnifies pre-existing social gradients in disease risk. These dynamics are particularly visible in rapidly growing cities, where low-income communities are often concentrated in flood-prone and heat-prone informal settlements. Water insecurity may necessitate household storage practices that favor *Aedes* mosquitos' proliferation, while overcrowding and poor drainage lead to increased mosquito breeding after heavy rainfall. In rural areas, marginalized and pastoral communities may face increased exposure through proximity to breeding habitats, seasonal mobility, and limited health facility access. Gender and age inequalities are also at play. Women, who often assume water collection and caregiving roles, and children, whose immunity and nutritional status may already be compromised, can experience higher disease burdens (24).

Over the last decade, African countries have adopted integrated approaches combining classic vector control with newer tools and climate-informed strategies (108, 109). This is illustrated in the growth of One Health initiatives across the continent, funded by both local and multinational institutions (Table 2). Focused initially on targeted responses to infectious disease outbreaks such as avian influenza and trypanosomiasis, One Health has since expanded to larger-scale interventions, engaging decision makers and community stakeholders through cross-sector collaborations. Such findings underscore the growing recognition of the systems-level interactions between animal, human, and ecological health within the healthcare, biomedical, and policymaking sectors (110). However, the practical implementation of these initiatives, focused to date on strategic planning and capacity-building, remains a challenge due to limited financing, institutional capacity, and health system coverage. Where inequalities remain unaddressed, even technically effective vector-control programs may deliver uneven benefits, reaching well-connected urban populations more rapidly than remote, displaced, or informal communities. Climate-informed vector control should be designed not only for entomological effectiveness, but also for social reach, affordability, and continuity in underserved settings.

**Table 2:** Select examples of collaborative climate-informed strategies and solutions implemented across Africa under the One Health approach, integrating environmental risk management with health systems strengthening and surveillance (128, 139–141).

xsInitiative	Countries involved	Year(s)	Key stakeholder(s)	Solution type	Solution(s) implemented
**Example 1**. Capacitating *One* Health in Eastern and Southern Africa (COHESA)	Botswana, Ethiopia, Kenya, Malawi, Mozambique, Namibia, Rwanda, Somalia, South Africa, Tanzania, Uganda, Zambia, Zimbabwe	2021–2025	Organization of African, Caribbean and Pacific States; International Livestock Research Institution; French Agricultural Research Center for International Development; International Service for the Acquisition of Agri-biotech Applications	Research and capacity-building	One Health governance, national data platforms, workshops and trainings
**Example 2**. Strengthening National Health Security through the *One* Health Approach	Cabo Verde	2023–present	The Pandemic Fund, Ministry of Health, Ministry of Environment, World Bank country office	Research and capacity-building	Laboratory surveillance, workshops and trainings
**Example 3**. Climate-Smart Agriculture (CSA) *One* Health Innovations	Ghana	2023–present	Accelerating Impacts of CGIAR Climate Research for Africa (AICCRA)	Adaptation and resilience	Pest management, soil management, nutrition, workshops and trainings
**Example 4**. Integrated Malaria Information System; DHIS2 Malaria Early Warning System	Mozambique	2022–present	Ministry of Health, Clinton Health Access Initiative, Wellcome Trust, HISP Center at University of Oslo	Surveillance and disease management	Early warning system, national data platforms, DHIS (district health information system)

Expanding climate-sensitive health frameworks is essential to enable effective responses to emerging global public health challenges. For instance, the effectiveness of biomedical tools relevant to malaria and arbovirus control in Africa, including vaccines, will depends on equitable coverage and sustained delivery systems capable of operating under climate stress and supply chain disruptions (111, 112). Hence, climate resilience needs to be integrated into the scientific development and implementation of malaria and arbovirus control strategies (2, 4). This implies combining innovation with equity-oriented delivery models, including strengthened primary care, community health workers, mobile surveillance, locally adapted risk communication, and targeted support for high-burden districts. Without addressing the broader socio-economic determinants of exposure and access, new technologies alone are unlikely to narrow climate-sensitive disease inequalities across the continent.

#### Limits and risks of policy transfer from advanced economies

One factor contributing to the difficulty faced by African countries in adopting the preparedness and mitigation plans that are modeled by advanced economies is the uneven integration of health literacy at the community level. This gap limits the effective incorporation of climate–health considerations into governance strategies (3). Where developmental conditions, including universal education access, digital connectivity, formal employment protections, stable housing, and comprehensive primary health-care coverage, are absent or incomplete, policy transfer becomes not only less effective, but also inequitable, as expectations clash with structural barriers to compliance.

In developed economies, preparedness measures typically are grounded on higher baseline levels of general literacy, digital access, institutional trust, and sustained public engagement. These conditions enable populations to interpret risk information and translate it into behavioral changes (113, 114). They are also reinforced by stronger welfare systems, insurance coverage, labor protections, and more reliable public services, which reduce the personal costs of adaptation. For example, workers may be better able to reduce outdoor activity during heatwaves when social protections, paid leave, or regulated occupational safeguards are available. By contrast, climate-health policies in many African countries are frequently framed in highly technical language and circulated primarily within restricted policy, diplomatic, and senior administrative circles. This practice limits the engagement of communities with the frontline professionals responsible for health system implementation. The resulting communication gap also reduces the alignment of defined policies with local knowledge, risk perceptions, and lived realities, which further diminishes their effectiveness (16). In many African settings, advice to remain indoors during extreme heat, eliminate mosquito breeding sites, or maintain food reserves may be unrealistic where shelter quality is poor, water storage is necessary, or incomes depend on daily outdoor work.

Similar limitations have been documented outside of the sphere of climate policy. In 2020, during the global coronavirus pandemic, COVID-19 control measures modeled on high-income lockdown approaches often proved socially unsustainable in low-income urban settings where large shares of the population depended on daily informal earnings and lacked income replacement mechanisms (115, 116). Likewise, water-intensive hygiene guidance targeted at improving sanitation in developing contexts is less feasible where reliable household water access is absent. These examples illustrate how technically sound policies may fail when socio-economic determinants and contexts are ignored.

From an equity perspective, such findings reveal why policy transfer may reproduce injustice when it fails to account for adaptation in local contexts. Climate preparedness frameworks that ignore pre-existing disparities risk shifting the burden of adaptation onto those already experiencing the highest exposure and the fewest resources (22). Rather than narrowing inequalities, these universalizing policies can deepen them by privileging populations with greater assets, connectivity, and institutional access. This pattern mirrors the broader pattern of uneven development across the African continent, where climate impacts interact with historical inequalities in infrastructure, service provision, and economic opportunity (16). Heat action plans provide another example of these dynamics in action. Urban warning systems copied from high-income settings may assume widespread access to air-conditioned indoor environments, and formal occupational protections, and real-time smartphone alerts. However, in many African cities, workers remain outdoors in informal labor markets, electricity supply may be unreliable, and digital alerts may not reach those at highest risk. Under such circumstances, policies may seem sound on paper while failing to protect exposed populations in practice (4).

By contrast, policy transfer can succeed when principles are adapted rather than copied. Community health worker models, decentralized vaccination outreach, and locally tailored early warning systems have shown stronger uptake in lower-income settings due to their integration into existing social networks and frontline service structures. Similarly, climate-smart agriculture programs that combine scientific forecasting with farmer knowledge and locally relevant crop choices have often outperformed standardized, externally-designed packages (117). These examples suggest that successful transfer depends on contextual translation, institutional fit, and co-ownership of imported policies.

### Health literacy, equity, and sustainability of climate actions

The capacity of individuals and communities to access, understand, appraise, and apply information to make informed decisions, commonly referred to as health literacy, is a critical determinant of climate resilience and adaptive capacity (118, 119). In the context of climate change, health literacy includes the social and institutional ability of communities to interpret risks collectively, negotiate locally feasible responses, and participate in decisions that affect their health and environment. This broader understanding is particularly relevant in Africa, where adaptation frequently depends on community-level action under conditions of constrained formal protection systems.

Populations with higher health literacy are more likely to respond appropriately to early warning systems, adopt preventive behaviors during heatwaves or disease outbreaks, and engage with health services in a timely manner (108, 120). In high-income settings, health literacy is embedded across education systems, primary health care, as well as occupational and communication infrastructures. This investment enables climate parameters, like temperature, air-quality, or vector alerts, to be translated into actionable behaviors, including increased hydration, elimination of mosquito breeding sites, and early-care seeking, which enhances the effectiveness of preparedness plans (4, 121). These systems are also supported by higher institutional trust, stronger public communication channels, and more consistent service delivery, allowing information to be converted into protective action with fewer structural barriers.

In many African countries, structural inequities such as lower average educational attainment, high linguistic diversity, digital divides and historical mistrust, which are rooted in colonial and post-colonial systems of governance, limit the rapid translation of climate–health information into actionable, protective measures (122). This gap is particularly relevant given the reliance on individual and household-driven coping strategies in response to climate hazards. For example, heat action plans that depend on individual outdoor labor reduction and fluid intake increase during extreme heat hours are less effective for workers who lack labor protections, income security, and/or access to locally reliable information (123). Similarly, vector-control strategies requiring household participation in eliminating breeding sites are undermined when water scarcity necessitates storage practices that increase exposure (101). Therefore, expanding climate–health preparedness in Africa requires leveraging health literacy as a core adaptation strategy. This implies a shift toward participatory approaches and recognizing communities as active agents of change (124, 125). Drawing on ethical frameworks of climate justice and Ubuntu, participatory approaches should not be viewed as optional additions to policy, but as core governance requirements. Ubuntu supports adaptation models in which communities are co-producers of resilience, involving community leaders, women's groups, youth networks, informal workers, and frontline health actors in the co-design, implementation, and evaluation of climate-health strategies. Such engagement increases legitimacy, trust, contextual relevance, and long-term policy adherence. Operationally, governments should institutionalize community participation through local adaptation committees, routine public consultations, multilingual risk communication platforms, and formal inclusion of community health workers and civil society organizations in national climate-health planning processes.

It is also important to highlight the following point. Preparedness models developed in high-income economies presume that individuals can act on information once it is provided. However, enabling action frequently requires addressing associated structural barriers, including proper access to water, sanitation and safe housing (11, 115). Populations that understand climate-health risks and the rationale for specific interventions are better equipped to participate in policy dialogue, assess strategy impact, improve initiatives and navigate misinformation (126). This capacity is especially important under conditions of climatic uncertainty, as social cohesion may influence the success of public health responses (127). Thus, the simple replication of externally-designed climate preparedness plans is not enough. There must also be an increase in international support to enhance technical assistance and community-led participation across the African continent, thus shifting the burden of climate adaptation away from those least responsible for climate change.

When structural barriers remain unaddressed, information alone risks transferring responsibility for adaptation onto vulnerable households. Hence, a first priority is the development of climate–health integrated surveillance systems that connect meteorological services, hydrological monitoring, agricultural early warning, vector surveillance, and routine health information systems. Such platforms would enable earlier detection of heatwaves, floods, drought-linked malnutrition, and climate-sensitive infectious disease outbreaks. A second priority is sustained investment in primary health infrastructure. Resilient primary care networks, laboratories, supply chains, cold-chain capacity, water and sanitation facilities, and decentralized emergency services are essential to translate climate information into life-saving action. Without these systems, warnings may be issued but remain clinically ineffective. Strengthening frontline health services also improves continuity of maternal care, vaccination, chronic disease treatment, and outbreak management during climate emergencies. A third priority concerns reform of climate finance mechanisms. Many current instruments remain difficult to access because of complex application procedures, fragmented funding windows, and burdensome reporting requirements. Simplifying access, expanding grant-based adaptation finance, supporting long-term institutional capacity, and channeling resources directly to subnational and community implementers would substantially improve implementation feasibility and equity. A fourth priority is embedding social protection into adaptation policy. Cash transfer systems, school feeding programs, labor safeguards during extreme heat events, crop and livestock insurance, and emergency nutrition support can buffer households from climate-related income and food insecurity while reducing negative coping strategies.

Hence, aligning climate–health interventions with local realities and strengthening health literacy is a prerequisite for equitable and sustainable climate governance in Africa (2, 3).

### From global ambition to local feasibility: health-centered climate actions implemented in Africa

Health-centered climate actions are the most effective adaptation strategies for African countries (2, 9), where adverse climate episodes often exacerbate illness and mortality rates (4, 6, 12). Tackling malnutrition, diarrheal disease, respiratory morbidity, and vector-borne infections as core aspects of national climate initiatives requires governments and stakeholders to institutionalize interventions, translate climate-health action plans into operational systems, scale climate-sensitive agriculture practices, and integrate vector control strategies into routine health care, sanitation, and environmental services (3, 12, 123, 128–130). Such measures will become more effective if adopted as routine, rather than activated mainly during episodes of emergency campaigns (131, 132).

The ambition of climate policy in Africa should be aligned with implementation feasibility. Many global standards presume the pre-existence of conditions for the effective operation of advanced regulatory frameworks (8, 11). However, in many African countries, these foundational systems often remain incomplete, among which are the availability of safe water and sanitation, solid waste management, urban drainage, reliable electricity, laboratory capacity, and functional disease surveillance systems. Hence, governments should pursue climate action targeted at investments to reduce population-level exposure to droughts, floods, heat and pollution and strengthen community-led participation and resilience (133, 134). Regulations on plastics, air quality, biodiversity protection, and indicator reporting become sustainable only once infrastructure systems are in place (135, 136).

Effective climate adaptation and health protection increasingly depend on integrated surveillance systems, capable to anticipate and respond proactively, rather than reactively, to environmental crises (116). African countries can achieve substantial gains by linking national meteorological services with public health institutions, vector control programs, agricultural monitoring systems, and water management authorities (108, 137). Such measures can improve the timeliness, efficiency, and equity of interventions under resource constraints (49, 105).

Taken together, a strategic shift in governance is required to reduce exposure and sensitivity to climate risks across the African continent, while delivering immediate gains in health and livelihoods. Prioritizing interventions with measurable population health impacts represents the most equitable, feasible, and pragmatic pathway toward climate-resilient development in the African context (10, 138).

## Conclusions

Africa stands at the frontline of the interconnected crises of climate change, biodiversity loss, pollution, food insecurity, and climate-sensitive disease. Expanding the scope of climate action to incorporate health, equity, and feasibility is essential for effective implementation of actionable strategies. These overlapping risks demonstrate that climate change in Africa is more than an environmental challenge. It is a profound ethical test of how global societies allocate responsibility, protection, and opportunity under conditions of shared planetary vulnerability.

Without structural reforms in global climate governance, international environmental standards risk remaining more aspirational than transformative in countries that face the greatest burdens of climate change and its health implications. From a climate justice perspective, such reforms are necessary to align responsibility for emissions with present obligations to finance adaptation, enable technology transfer, strengthen health systems, and expand decision-making power for regions facing disproportionate harm. Equity in this sense requires more than financial redistribution. It requires redesigning governance systems so that feasibility, vulnerability, and public health outcomes carry equal weight alongside mitigation targets. By grounding climate policy in global health principles and lived realities, Africa will move toward a more sustainable future, for all.
